# Tetra­kis(μ-2-methyl­benzoato-κ^2^ *O*:*O*′)bis­[(methanol-κ*O*)copper(II)]

**DOI:** 10.1107/S1600536810013322

**Published:** 2010-04-17

**Authors:** Muhammad Danish, Iram Saleem, M. Nawaz Tahir, Nazir Ahmad, Abdur Rauf Raza

**Affiliations:** a Department of Chemistry, University of Sargodha, Sargodha, Pakistan; b Department of Physics, University of Sargodha, Sargodha, Pakistan

## Abstract

In the title compound, [Cu_2_(C_8_H_7_O_2_)_4_(CH_3_OH)_2_], the Cu—O bond distances are in the range 1.943 (2)–2.149 (2) Å within a sligthly distorted square-pyramidal coordination. The Cu⋯Cu separation is 2.5912 (4) Å. In the crystal, the mol­ecules are linked into polymeric chains propagating in [001] by inter­molecular O—H⋯O hydrogen bonds and C—H⋯π inter­actions.

## Related literature

For our work on the synthesis of various metal complexes of 2-methyl­benzoic acid, see: Danish *et al.* (2010 ▶). For related structures, see: Kabbani *et al.* (2004 ▶); Rao *et al.* (1983 ▶); Sunil *et al.* (2008 ▶); Xin & Liu (2008 ▶).=
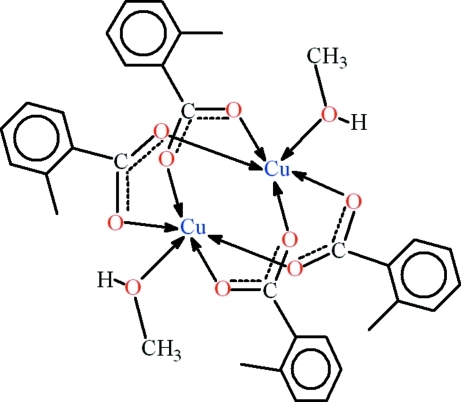


## Experimental

### 

#### Crystal data

[Cu_2_(C_8_H_7_O_2_)_4_(CH_4_O)_2_]
*M* *_r_* = 731.71
Triclinic, 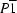

*a* = 10.7474 (9) Å
*b* = 12.1403 (11) Å
*c* = 14.1709 (11) Å
α = 113.805 (2)°
β = 91.096 (3)°
γ = 93.238 (3)°
*V* = 1687.2 (2) Å^3^
*Z* = 2
Mo *K*α radiation
μ = 1.32 mm^−1^
*T* = 296 K
0.30 × 0.14 × 0.08 mm

#### Data collection

Bruker Kappa APEXII CCD diffractometer
Absorption correction: multi-scan (*SADABS*; Bruker, 2005 ▶) *T*_min_ = 0.804, *T*_max_ = 0.898
27787 measured reflections
8232 independent reflections
5004 reflections with *I* > 2σ(*I*)
*R*_int_ = 0.047

#### Refinement

*R*[*F* ^2^ > 2σ(*F* ^2^)] = 0.043
*wR*(*F* ^2^) = 0.098
*S* = 1.02
8232 reflections
427 parameters
H atoms treated by a mixture of independent and constrained refinement
Δρ_max_ = 0.30 e Å^−3^
Δρ_min_ = −0.31 e Å^−3^

### 

Data collection: *APEX2* (Bruker, 2007 ▶); cell refinement: *SAINT* (Bruker, 2007 ▶); data reduction: *SAINT*; program(s) used to solve structure: *SHELXS97* (Sheldrick, 2008 ▶); program(s) used to refine structure: *SHELXL97* (Sheldrick, 2008 ▶); molecular graphics: *ORTEP-3 for Windows* (Farrugia, 1997 ▶) and *PLATON* (Spek, 2009 ▶); software used to prepare material for publication: *WinGX* (Farrugia, 1999 ▶) and *PLATON*.

## Supplementary Material

Crystal structure: contains datablocks global, I. DOI: 10.1107/S1600536810013322/bq2206sup1.cif

Structure factors: contains datablocks I. DOI: 10.1107/S1600536810013322/bq2206Isup2.hkl

Additional supplementary materials:  crystallographic information; 3D view; checkCIF report

## Figures and Tables

**Table 1:** Hydrogen-bond geometry (Å, °) *Cg*1 and *Cg*2 are the centroids of C2–C7 and C28–C33 rings, respectively.

*D*—H⋯*A*	*D*—H	H⋯*A*	*D*⋯*A*	*D*—H⋯*A*
O5—H5*A*⋯O7^i^	0.70 (3)	2.11 (3)	2.793 (3)	167 (4)
O6—H6*A*⋯O2^ii^	0.72 (3)	2.10 (3)	2.812 (2)	174 (4)
C18—H18*A*⋯*Cg*1^ii^	0.96	2.98	3.749 (4)	137.00
C23—H23⋯*Cg*1^iii^	0.93	2.87	3.735 (4)	154.00
C13—H13⋯*Cg*2^iv^	0.93	2.97	3.877 (4)	165.00

Symmetry codes: (i) 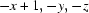; (ii) 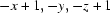; (iii) 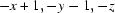; (iv) 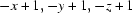.

**Fig. 1.**
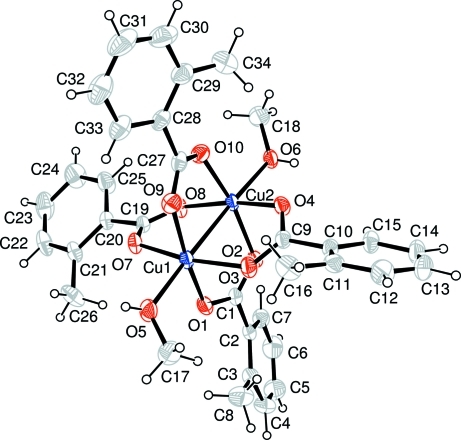
View of the title compound with the atom numbering scheme. The thermal ellipsoids are drawn at the 30% probability level.

**Fig. 2.**
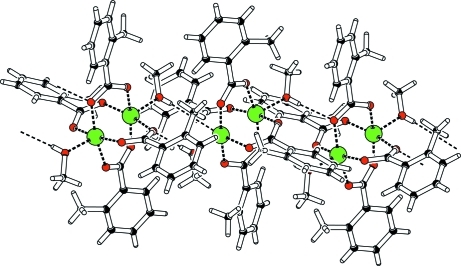
The partial packing (PLATON; Spek, 2009) which shows that molecules form polymeric sheets due to the intermolecular H-bondings.

## supplementary crystallographic information

### Comment

In continuation to synthesize various metal complexes of 2-methylbenzoic acid (Danish *et al.*, 2010), the title compound (I) (Fig. 1) is being reported here.

The crystal structure of (II) Tetrakis(µ-2-methylbenzoato-O,*O*') -bis(2-methylbenzoic acid-O)-di-copper(ii) (Sunil *et al.*, 2008) has been reported. The title compound (I) differs from (II) due to the bonding of methanol at apical positions instead of *o*-toluic acid. The crystal structures of (III) Tetrakis(µ-2-anilinobenzoato)-bis(methanol-copper(ii)) (Xin & Liu, 2008), (IV) Tetrakis(µ-4-chloro-3-nitrobenzoato)-bis(methanol)-di-copper(ii) (Kabbani *et al.*, 2004) and (V) Tetrakis(µ-acetato)-bis(methanol)-di-copper(ii) (Rao *et al.*, 1983) have also been reported which have similar environments around Cu-atoms as in (I).

Although the space group is centrosymmetric but the molecules of (I) are not centrosymmetric. The C-atoms of 2-methylbenzoato A (C1—C8), B (C9—C16), C (C19—C26) and D (C27—C34) are planar with maximum r. m. s. deviations of 0.0148, 0.0278, 0.0111 & 0.0127 Å respectively, from the mean square planes. The carboxylato groups E (O1/C1/O2), F (O3/C9/O4), G (O7/C19/O8) and H (O9/C27/O10) are of course planar. The dihedral angle between A/E, B/F, C/G and D/H is 35.01 (23), 40.19 (20), 36.09 (25) and 27.16 (33)° respectively. The oppositely bonded 2-methylbenzoato groups with Cu-atoms make dihedral angles A/C 75.77 (7)° and B/D 86.29 (7)° which confirms that asymmetric units cannot be centrosymmetric. The coordination of Cu–O bond distances range 1.943 (2)–2.149 (2) Å [Table 1] and the separation between Cu to Cu is 2.5912 (4) Å. The molecules are stabilized in the form of polymeric chains due to intermolecular H-bondings and C–H···π interactions (Table 2, Fig. 2).

### Experimental

The sodium salt of 2-methylbenzoic acid (1 g, 6.32 mmol) was dissolved in 20 ml distilled water. Cu_2_So_4_.5H_2_O (0.789 g, 3.16 mmol) was separately dissolved in 20 ml distilled water. The former solution was slowly added to the later under continuous stirring with the formation of greenish precipitates. The reaction mixture was refluxed for 3 h and cooled to room temperature. The residue of filtration was crystallized in methanol:water (1:1). Green plate like crystals were obtained after 48 h.

### Refinement

The coordinates of H5A and H6A were refined. The H-atoms were positioned geometrically (C–H = 0.93–0.96 Å) and refined as riding with *U*_iso_(H) = x*U*_eq_(C, O), where x = 1.2 for aryl & hydroxy and 1.5 for methyl H-atoms.

### Crystal data

**Table:**

[Cu_2_(C_8_H_7_O_2_)_4_(CH_4_O)_2_]	*Z* = 2
*M**_r_* = 731.71	*F*(000) = 756
Triclinic, *P*1	*D*_x_ = 1.440 Mg m^−^^3^
Hall symbol: -P 1	Mo *K*α radiation, λ = 0.71073 Å
*a* = 10.7474 (9) Å	Cell parameters from 5004 reflections
*b* = 12.1403 (11) Å	θ = 1.9–28.3°
*c* = 14.1709 (11) Å	µ = 1.32 mm^−^^1^
α = 113.805 (2)°	*T* = 296 K
β = 91.096 (3)°	Plate, green
γ = 93.238 (3)°	0.30 × 0.14 × 0.08 mm
*V* = 1687.2 (2) Å^3^	

### Data collection

**Table:**

Bruker Kappa APEXII CCD diffractometer	8232 independent reflections
Radiation source: fine-focus sealed tube	5004 reflections with *I* > 2σ(*I*)
graphite	*R*_int_ = 0.047
Detector resolution: 7.40 pixels mm^-1^	θ_max_ = 28.3°, θ_min_ = 1.9°
ω scans	*h* = −14→14
Absorption correction: multi-scan (*SADABS*; Bruker, 2005)	*k* = −15→16
*T*_min_ = 0.804, *T*_max_ = 0.898	*l* = −18→16
27787 measured reflections	

### Refinement

**Table:**

Refinement on *F*^2^	Primary atom site location: structure-invariant direct methods
Least-squares matrix: full	Secondary atom site location: difference Fourier map
*R*[*F*^2^ > 2σ(*F*^2^)] = 0.043	Hydrogen site location: inferred from neighbouring sites
*wR*(*F*^2^) = 0.098	H atoms treated by a mixture of independent and constrained refinement
*S* = 1.02	*w* = 1/[σ^2^(*F*_o_^2^) + (0.0377*P*)^2^] where *P* = (*F*_o_^2^ + 2*F*_c_^2^)/3
8232 reflections	(Δ/σ)_max_ = 0.001
427 parameters	Δρ_max_ = 0.30 e Å^−^^3^
0 restraints	Δρ_min_ = −0.31 e Å^−^^3^

### Special details

**Table:**

Geometry. Bond distances, angles etc. have been calculated using the rounded fractional coordinates. All su's are estimated from the variances of the (full) variance-covariance matrix. The cell esds are taken into account in the estimation of distances, angles and torsion angles
Refinement. Refinement of *F*^2^ against ALL reflections. The weighted *R*-factor *wR* and goodness of fit *S* are based on *F*^2^, conventional *R*-factors *R* are based on *F*, with *F* set to zero for negative *F*^2^. The threshold expression of *F*^2^ > σ(*F*^2^) is used only for calculating *R*-factors(gt) *etc*. and is not relevant to the choice of reflections for refinement. *R*-factors based on *F*^2^ are statistically about twice as large as those based on *F*, and *R*- factors based on ALL data will be even larger.

### Fractional atomic coordinates and isotropic or equivalent isotropic displacement parameters (Å^2^)

**Table:**

	*x*	*y*	*z*	*U*_iso_*/*U*_eq_	
Cu1	0.46596 (3)	0.03113 (3)	0.18544 (2)	0.0408 (1)	
Cu2	0.58723 (3)	−0.00162 (3)	0.33076 (2)	0.0375 (1)	
O1	0.33402 (17)	−0.07793 (17)	0.20094 (13)	0.0491 (7)	
O2	0.42139 (16)	−0.07056 (17)	0.34848 (12)	0.0455 (6)	
O3	0.40272 (17)	0.16577 (17)	0.30278 (13)	0.0491 (7)	
O4	0.53752 (17)	0.15696 (16)	0.42153 (12)	0.0455 (7)	
O5	0.3622 (2)	0.0618 (2)	0.06817 (14)	0.0594 (8)	
O6	0.68139 (18)	−0.02251 (19)	0.45600 (13)	0.0480 (7)	
O7	0.54674 (17)	−0.11478 (16)	0.09317 (12)	0.0457 (6)	
O8	0.61682 (17)	−0.15929 (16)	0.22255 (12)	0.0475 (7)	
O9	0.61515 (18)	0.12928 (17)	0.18536 (14)	0.0519 (8)	
O10	0.73055 (16)	0.07076 (18)	0.28750 (14)	0.0498 (7)	
C1	0.3345 (3)	−0.1038 (3)	0.27850 (19)	0.0434 (10)	
C2	0.2270 (3)	−0.1791 (3)	0.29092 (18)	0.0456 (10)	
C3	0.1056 (3)	−0.1725 (3)	0.2599 (2)	0.0603 (11)	
C4	0.0122 (3)	−0.2479 (4)	0.2774 (3)	0.0803 (14)	
C5	0.0399 (4)	−0.3253 (4)	0.3202 (3)	0.0863 (17)	
C6	0.1585 (4)	−0.3326 (3)	0.3493 (3)	0.0762 (16)	
C7	0.2525 (3)	−0.2584 (3)	0.3367 (2)	0.0558 (11)	
C8	0.0690 (3)	−0.0852 (4)	0.2159 (3)	0.0924 (18)	
C9	0.4503 (3)	0.2038 (2)	0.39346 (19)	0.0415 (9)	
C10	0.3955 (2)	0.3091 (3)	0.4757 (2)	0.0425 (9)	
C11	0.3564 (3)	0.4071 (3)	0.4575 (2)	0.0531 (11)	
C12	0.3038 (3)	0.4971 (3)	0.5388 (3)	0.0734 (12)	
C13	0.2873 (3)	0.4904 (3)	0.6324 (3)	0.0799 (16)	
C14	0.3276 (3)	0.3954 (3)	0.6496 (2)	0.0668 (11)	
C15	0.3833 (3)	0.3061 (3)	0.5714 (2)	0.0533 (11)	
C16	0.3751 (3)	0.4205 (3)	0.3569 (2)	0.0694 (12)	
C17	0.2436 (3)	0.1088 (3)	0.0821 (3)	0.0784 (14)	
C18	0.8080 (3)	−0.0418 (3)	0.4644 (3)	0.0784 (14)	
C19	0.5962 (2)	−0.1844 (3)	0.12745 (18)	0.0411 (9)	
C20	0.6378 (3)	−0.3025 (3)	0.05359 (19)	0.0426 (9)	
C21	0.5751 (3)	−0.3735 (3)	−0.0404 (2)	0.0530 (11)	
C22	0.6264 (4)	−0.4802 (3)	−0.1010 (2)	0.0758 (13)	
C23	0.7336 (4)	−0.5161 (3)	−0.0737 (3)	0.0876 (18)	
C24	0.7955 (4)	−0.4456 (3)	0.0187 (3)	0.0889 (17)	
C25	0.7459 (3)	−0.3403 (3)	0.0815 (2)	0.0686 (11)	
C26	0.4549 (3)	−0.3410 (3)	−0.0754 (2)	0.0814 (16)	
C27	0.7166 (3)	0.1278 (3)	0.2308 (2)	0.0465 (10)	
C28	0.8269 (3)	0.1984 (3)	0.2150 (2)	0.0500 (11)	
C29	0.9268 (3)	0.2476 (3)	0.2875 (3)	0.0651 (12)	
C30	1.0209 (3)	0.3152 (3)	0.2657 (3)	0.0907 (18)	
C31	1.0204 (4)	0.3331 (4)	0.1767 (4)	0.0993 (19)	
C32	0.9236 (4)	0.2825 (4)	0.1051 (3)	0.0873 (17)	
C33	0.8266 (3)	0.2161 (3)	0.1243 (3)	0.0690 (12)	
C34	0.9365 (3)	0.2357 (4)	0.3889 (3)	0.0965 (19)	
H4	−0.07050	−0.24412	0.25903	0.0966*	
H5	−0.02370	−0.37447	0.32978	0.1035*	
H5A	0.387 (3)	0.065 (3)	0.024 (2)	0.0713*	
H6	0.17652	−0.38726	0.37758	0.0911*	
H6A	0.654 (3)	0.005 (3)	0.505 (2)	0.0576*	
H7	0.33377	−0.26120	0.35883	0.0670*	
H8A	0.09672	−0.11072	0.14671	0.1379*	
H8B	0.10695	−0.00639	0.25763	0.1379*	
H8C	−0.02007	−0.08249	0.21503	0.1379*	
H12	0.27881	0.56437	0.52971	0.0880*	
H13	0.24844	0.55104	0.68415	0.0959*	
H14	0.31764	0.39110	0.71309	0.0798*	
H15	0.41343	0.24222	0.58320	0.0639*	
H16A	0.37026	0.50353	0.36801	0.1038*	
H16B	0.31144	0.37174	0.30660	0.1038*	
H16C	0.45559	0.39470	0.33218	0.1038*	
H17A	0.20305	0.09234	0.13523	0.1172*	
H17B	0.19406	0.07159	0.01864	0.1172*	
H17C	0.25292	0.19441	0.10173	0.1172*	
H18A	0.85558	0.03454	0.49473	0.1179*	
H18B	0.83704	−0.08773	0.39700	0.1179*	
H18C	0.81790	−0.08536	0.50718	0.1179*	
H22	0.58535	−0.52973	−0.16344	0.0910*	
H23	0.76504	−0.58800	−0.11737	0.1052*	
H24	0.86927	−0.46871	0.03826	0.1070*	
H25	0.78647	−0.29294	0.14474	0.0825*	
H26A	0.40631	−0.41338	−0.11865	0.1221*	
H26B	0.47288	−0.29272	−0.11359	0.1221*	
H26C	0.40881	−0.29619	−0.01627	0.1221*	
H30	1.08722	0.34991	0.31370	0.1087*	
H31	1.08520	0.37933	0.16478	0.1188*	
H32	0.92324	0.29288	0.04354	0.1050*	
H33	0.76043	0.18283	0.07590	0.0826*	
H34A	0.99931	0.29385	0.43328	0.1448*	
H34B	0.95867	0.15587	0.37725	0.1448*	
H34C	0.85763	0.24976	0.42093	0.1448*	

### Atomic displacement parameters (Å^2^)

**Table:**

	*U*^11^	*U*^22^	*U*^33^	*U*^12^	*U*^13^	*U*^23^
Cu1	0.0499 (2)	0.0448 (2)	0.0270 (2)	0.0077 (2)	0.0030 (1)	0.0133 (2)
Cu2	0.0442 (2)	0.0408 (2)	0.0248 (2)	0.0041 (2)	0.0020 (1)	0.0104 (2)
O1	0.0550 (12)	0.0557 (14)	0.0322 (9)	−0.0021 (10)	−0.0024 (9)	0.0141 (10)
O2	0.0463 (11)	0.0563 (13)	0.0301 (9)	−0.0044 (9)	−0.0017 (8)	0.0149 (9)
O3	0.0596 (12)	0.0473 (13)	0.0354 (10)	0.0151 (10)	0.0025 (9)	0.0101 (9)
O4	0.0522 (12)	0.0411 (12)	0.0350 (10)	0.0080 (9)	−0.0018 (8)	0.0066 (9)
O5	0.0662 (14)	0.0818 (17)	0.0404 (12)	0.0200 (12)	0.0083 (10)	0.0331 (12)
O6	0.0541 (13)	0.0587 (14)	0.0286 (10)	0.0147 (10)	0.0019 (9)	0.0135 (10)
O7	0.0634 (12)	0.0453 (12)	0.0285 (9)	0.0133 (10)	0.0071 (9)	0.0135 (9)
O8	0.0696 (13)	0.0420 (12)	0.0276 (9)	0.0116 (10)	0.0033 (9)	0.0096 (9)
O9	0.0546 (13)	0.0535 (14)	0.0531 (12)	0.0057 (10)	0.0060 (10)	0.0270 (11)
O10	0.0459 (11)	0.0567 (14)	0.0509 (11)	0.0039 (10)	0.0076 (9)	0.0258 (11)
C1	0.0482 (17)	0.0451 (19)	0.0301 (14)	0.0040 (14)	0.0045 (13)	0.0079 (14)
C2	0.0458 (18)	0.0491 (19)	0.0287 (13)	−0.0008 (14)	0.0008 (12)	0.0027 (14)
C3	0.056 (2)	0.070 (2)	0.0434 (17)	0.0019 (18)	−0.0024 (15)	0.0117 (17)
C4	0.048 (2)	0.108 (3)	0.070 (2)	−0.015 (2)	−0.0107 (18)	0.024 (2)
C5	0.069 (3)	0.097 (3)	0.081 (3)	−0.021 (2)	0.006 (2)	0.027 (3)
C6	0.089 (3)	0.063 (3)	0.073 (2)	−0.007 (2)	0.012 (2)	0.025 (2)
C7	0.061 (2)	0.052 (2)	0.0481 (17)	−0.0054 (17)	0.0063 (15)	0.0149 (16)
C8	0.065 (2)	0.122 (4)	0.094 (3)	0.017 (2)	−0.009 (2)	0.047 (3)
C9	0.0457 (17)	0.0377 (18)	0.0358 (14)	−0.0018 (14)	0.0059 (13)	0.0100 (14)
C10	0.0432 (16)	0.0363 (17)	0.0404 (15)	0.0014 (13)	0.0052 (12)	0.0077 (14)
C11	0.0484 (18)	0.046 (2)	0.0548 (18)	0.0029 (15)	−0.0038 (14)	0.0102 (16)
C12	0.080 (2)	0.054 (2)	0.078 (2)	0.0237 (19)	0.000 (2)	0.016 (2)
C13	0.077 (3)	0.064 (3)	0.066 (2)	0.021 (2)	0.0135 (19)	−0.010 (2)
C14	0.077 (2)	0.057 (2)	0.0482 (18)	0.0014 (19)	0.0166 (16)	0.0025 (18)
C15	0.066 (2)	0.0400 (19)	0.0446 (16)	0.0025 (15)	0.0129 (14)	0.0072 (15)
C16	0.081 (2)	0.056 (2)	0.074 (2)	0.0041 (19)	−0.0089 (19)	0.0300 (19)
C17	0.072 (2)	0.107 (3)	0.075 (2)	0.029 (2)	0.0098 (19)	0.053 (2)
C18	0.062 (2)	0.092 (3)	0.070 (2)	0.025 (2)	−0.0013 (18)	0.019 (2)
C19	0.0437 (16)	0.0451 (18)	0.0315 (14)	0.0013 (14)	0.0030 (12)	0.0125 (14)
C20	0.0566 (18)	0.0373 (17)	0.0318 (13)	0.0053 (14)	0.0062 (13)	0.0115 (13)
C21	0.075 (2)	0.044 (2)	0.0341 (15)	−0.0026 (16)	−0.0017 (14)	0.0108 (15)
C22	0.121 (3)	0.054 (2)	0.0358 (16)	0.002 (2)	0.0013 (19)	0.0018 (17)
C23	0.135 (4)	0.059 (3)	0.060 (2)	0.041 (3)	0.021 (2)	0.010 (2)
C24	0.102 (3)	0.076 (3)	0.078 (3)	0.046 (2)	0.006 (2)	0.015 (2)
C25	0.081 (2)	0.065 (2)	0.0493 (18)	0.025 (2)	−0.0042 (17)	0.0100 (18)
C26	0.093 (3)	0.069 (3)	0.062 (2)	−0.007 (2)	−0.032 (2)	0.009 (2)
C27	0.055 (2)	0.0388 (18)	0.0419 (15)	0.0107 (15)	0.0153 (14)	0.0109 (14)
C28	0.0520 (19)	0.0368 (18)	0.0594 (18)	0.0067 (15)	0.0205 (15)	0.0165 (16)
C29	0.055 (2)	0.052 (2)	0.078 (2)	0.0041 (17)	0.0145 (18)	0.0153 (19)
C30	0.061 (2)	0.074 (3)	0.124 (4)	−0.011 (2)	0.016 (2)	0.028 (3)
C31	0.084 (3)	0.073 (3)	0.140 (4)	−0.006 (2)	0.045 (3)	0.042 (3)
C32	0.103 (3)	0.076 (3)	0.098 (3)	0.008 (3)	0.040 (3)	0.049 (3)
C33	0.071 (2)	0.065 (2)	0.079 (2)	0.0077 (19)	0.0254 (19)	0.036 (2)
C34	0.082 (3)	0.107 (4)	0.084 (3)	−0.014 (2)	−0.011 (2)	0.025 (3)

### Geometric parameters (Å, °)

**Table:**

Cu1—O1	1.961 (2)	C23—C24	1.374 (6)
Cu1—O3	1.9681 (19)	C24—C25	1.374 (5)
Cu1—O5	2.149 (2)	C27—C28	1.497 (5)
Cu1—O7	1.9871 (19)	C28—C33	1.386 (5)
Cu1—O9	1.943 (2)	C28—C29	1.398 (5)
Cu1—Cu2	2.5912 (4)	C29—C34	1.503 (6)
Cu2—O2	1.9896 (19)	C29—C30	1.384 (5)
Cu2—O4	1.9468 (19)	C30—C31	1.364 (7)
Cu2—O6	2.1358 (19)	C31—C32	1.369 (6)
Cu2—O8	1.9582 (18)	C32—C33	1.380 (6)
Cu2—O10	1.959 (2)	C4—H4	0.9300
O1—C1	1.259 (4)	C5—H5	0.9300
O2—C1	1.272 (3)	C6—H6	0.9300
O3—C9	1.263 (3)	C7—H7	0.9300
O4—C9	1.261 (4)	C8—H8A	0.9600
O5—C17	1.411 (4)	C8—H8B	0.9600
O6—C18	1.405 (4)	C8—H8C	0.9600
O7—C19	1.267 (4)	C12—H12	0.9300
O8—C19	1.268 (3)	C13—H13	0.9300
O9—C27	1.260 (4)	C14—H14	0.9300
O10—C27	1.266 (4)	C15—H15	0.9300
O5—H5A	0.70 (3)	C16—H16A	0.9600
O6—H6A	0.72 (3)	C16—H16B	0.9600
C1—C2	1.490 (5)	C16—H16C	0.9600
C2—C7	1.396 (5)	C17—H17A	0.9600
C2—C3	1.386 (5)	C17—H17B	0.9600
C3—C4	1.413 (6)	C17—H17C	0.9600
C3—C8	1.496 (6)	C18—H18A	0.9600
C4—C5	1.351 (7)	C18—H18B	0.9600
C5—C6	1.351 (6)	C18—H18C	0.9600
C6—C7	1.378 (5)	C22—H22	0.9300
C9—C10	1.499 (4)	C23—H23	0.9300
C10—C11	1.400 (5)	C24—H24	0.9300
C10—C15	1.380 (4)	C25—H25	0.9300
C11—C16	1.515 (4)	C26—H26A	0.9600
C11—C12	1.385 (5)	C26—H26B	0.9600
C12—C13	1.375 (6)	C26—H26C	0.9600
C13—C14	1.363 (5)	C30—H30	0.9300
C14—C15	1.375 (4)	C31—H31	0.9300
C19—C20	1.493 (4)	C32—H32	0.9300
C20—C25	1.381 (5)	C33—H33	0.9300
C20—C21	1.394 (4)	C34—H34A	0.9600
C21—C26	1.504 (5)	C34—H34B	0.9600
C21—C22	1.386 (5)	C34—H34C	0.9600
C22—C23	1.358 (6)		
			
Cu1···O2	3.0610 (19)	C2···C18^ii^	3.466 (5)
Cu1···O4	3.1243 (16)	C6···C34^ii^	3.598 (6)
Cu1···O8	3.102 (2)	C8···O1	2.859 (4)
Cu1···O10	3.0880 (18)	C9···C1	3.555 (4)
Cu1···O5^i^	3.849 (2)	C11···C11^iii^	3.598 (5)
Cu2···O7	3.0911 (16)	C15···C18^ii^	3.561 (5)
Cu2···O1	3.1299 (18)	C15···O6^ii^	3.336 (4)
Cu2···O3	3.049 (2)	C16···O3	2.905 (4)
Cu2···O9	3.078 (2)	C17···O7^i^	3.405 (4)
Cu1···H5A^i^	3.20 (3)	C17···C31^iv^	3.589 (6)
Cu2···H6A^ii^	3.53 (3)	C18···C15^ii^	3.561 (5)
Cu2···H15^ii^	3.5900	C18···C2^ii^	3.466 (5)
O1···Cu2	3.1299 (18)	C19···C1	3.507 (4)
O1···O3	2.765 (3)	C26···O7	2.921 (4)
O1···O5	3.008 (3)	C31···C17^v^	3.589 (6)
O1···O7	2.731 (3)	C34···O10	2.834 (4)
O1···C8	2.859 (4)	C34···C6^ii^	3.598 (6)
O1···C19	3.183 (3)	C1···H6A^ii^	2.80 (3)
O2···C9	3.124 (3)	C1···H8B	2.8400
O2···O4	2.741 (3)	C2···H18A^ii^	3.0100
O2···Cu1	3.0610 (19)	C2···H6A^ii^	3.01 (3)
O2···O3	3.202 (3)	C2···H23^vi^	2.9100
O2···O6	3.067 (3)	C4···H18B^iv^	2.8400
O2···O8	2.758 (2)	C4···H25^iv^	2.9300
O2···O6^ii^	2.812 (2)	C6···H34C^ii^	3.0100
O3···C1	3.190 (4)	C7···H23^vi^	2.9000
O3···O9	2.800 (3)	C7···H6A^ii^	3.08 (3)
O3···Cu2	3.049 (2)	C9···H16C	2.7800
O3···C16	2.905 (4)	C12···H16C^iii^	3.0400
O3···O5	3.055 (2)	C13···H16C^iii^	2.9600
O3···O1	2.765 (3)	C19···H5A^i^	3.04 (3)
O3···O2	3.202 (3)	C19···H26C	2.7100
O3···C17	3.331 (4)	C25···H4^v^	2.9600
O4···O6	2.930 (3)	C26···H33^i^	3.0900
O4···C27	3.262 (3)	C27···H34C	2.8500
O4···O2	2.741 (3)	C28···H13^iii^	2.9600
O4···Cu1	3.1243 (16)	C29···H13^iii^	3.0700
O4···O10	2.793 (2)	C31···H17C^v^	3.0600
O5···Cu1^i^	3.849 (2)	H4···C25^iv^	2.9600
O5···O7^i^	2.793 (3)	H4···H8C	2.3300
O5···O7	3.118 (3)	H4···H18B^iv^	2.3900
O5···O9	3.049 (3)	H4···H25^iv^	2.0900
O5···O3	3.055 (2)	H5A···Cu1^i^	3.20 (3)
O5···O1	3.008 (3)	H5A···O7^i^	2.11 (3)
O6···O4	2.930 (3)	H5A···C19^i^	3.04 (3)
O6···C15^ii^	3.336 (4)	H6···H16A^vii^	2.5100
O6···O2^ii^	2.812 (2)	H6A···Cu2^ii^	3.53 (3)
O6···O2	3.067 (3)	H6A···O2^ii^	2.10 (3)
O6···O8	3.087 (2)	H6A···C1^ii^	2.80 (3)
O6···O10	3.070 (3)	H6A···C2^ii^	3.01 (3)
O7···O5^i^	2.793 (3)	H6A···C7^ii^	3.08 (3)
O7···O1	2.731 (3)	H7···O2	2.5100
O7···Cu2	3.0911 (16)	H8A···O1	2.6100
O7···C27	3.229 (4)	H8B···O1	2.6700
O7···C26	2.921 (4)	H8B···C1	2.8400
O7···C17^i^	3.405 (4)	H8C···H4	2.3300
O7···O9	2.760 (3)	H12···H16A	2.3600
O7···O5	3.118 (3)	H13···C28^iii^	2.9600
O8···C1	3.191 (4)	H13···C29^iii^	3.0700
O8···O6	3.087 (2)	H15···O4	2.5400
O8···O2	2.758 (2)	H15···Cu2^ii^	3.5900
O8···O10	2.764 (3)	H15···O6^ii^	2.6300
O8···Cu1	3.102 (2)	H16A···H6^viii^	2.5100
O9···O7	2.760 (3)	H16A···H12	2.3600
O9···Cu2	3.078 (2)	H16B···O3	2.7200
O9···O5	3.049 (3)	H16B···H30^iv^	2.4200
O9···C9	3.299 (3)	H16C···O3	2.6600
O9···O3	2.800 (3)	H16C···C9	2.7800
O10···C18	3.414 (4)	H16C···C12^iii^	3.0400
O10···O4	2.793 (2)	H16C···C13^iii^	2.9600
O10···Cu1	3.0880 (18)	H17C···C31^iv^	3.0600
O10···C19	3.244 (4)	H18A···C2^ii^	3.0100
O10···C34	2.834 (4)	H18B···C4^v^	2.8400
O10···O8	2.764 (3)	H18B···H4^v^	2.3900
O10···O6	3.070 (3)	H22···H26A	2.3900
O1···H8A	2.6100	H23···C2^vi^	2.9100
O1···H8B	2.6700	H23···C7^vi^	2.9000
O2···H7	2.5100	H25···O8	2.4800
O2···H6A^ii^	2.10 (3)	H25···C4^v^	2.9300
O3···H16C	2.6600	H25···H4^v^	2.0900
O3···H16B	2.7200	H26A···H22	2.3900
O4···H6A	2.89 (4)	H26B···O7	2.9100
O4···H15	2.5400	H26B···O9^i^	2.7700
O6···H15^ii^	2.6300	H26C···O7	2.5000
O7···H5A^i^	2.11 (3)	H26C···C19	2.7100
O7···H26C	2.5000	H30···H16B^v^	2.4200
O7···H26B	2.9100	H30···H34A	2.2700
O8···H25	2.4800	H33···O9	2.4600
O9···H33	2.4600	H33···C26^i^	3.0900
O9···H26B^i^	2.7700	H34A···H30	2.2700
O10···H34B	2.6900	H34B···O10	2.6900
O10···H34C	2.5300	H34C···O10	2.5300
C1···C9	3.555 (4)	H34C···C27	2.8500
C1···C19	3.507 (4)	H34C···C6^ii^	3.0100
			
O1—Cu1—O3	89.46 (8)	C29—C28—C33	119.7 (3)
O1—Cu1—O5	93.97 (8)	C27—C28—C29	123.1 (3)
O1—Cu1—O7	87.51 (8)	C28—C29—C30	117.6 (3)
O1—Cu1—O9	169.65 (8)	C28—C29—C34	124.5 (3)
O3—Cu1—O5	95.69 (8)	C30—C29—C34	117.9 (3)
O3—Cu1—O7	166.39 (8)	C29—C30—C31	122.7 (4)
O3—Cu1—O9	91.44 (8)	C30—C31—C32	119.4 (4)
O5—Cu1—O7	97.76 (8)	C31—C32—C33	120.0 (4)
O5—Cu1—O9	96.20 (9)	C28—C33—C32	120.6 (3)
O7—Cu1—O9	89.21 (8)	C3—C4—H4	119.00
O2—Cu2—O4	88.25 (8)	C5—C4—H4	119.00
O2—Cu2—O6	95.98 (8)	C4—C5—H5	119.00
O2—Cu2—O8	88.61 (8)	C6—C5—H5	119.00
O2—Cu2—O10	166.98 (8)	C5—C6—H6	120.00
O4—Cu2—O6	91.63 (8)	C7—C6—H6	120.00
O4—Cu2—O8	170.33 (7)	C2—C7—H7	120.00
O4—Cu2—O10	91.27 (8)	C6—C7—H7	120.00
O6—Cu2—O8	97.79 (8)	C3—C8—H8A	109.00
O6—Cu2—O10	97.04 (8)	C3—C8—H8B	109.00
O8—Cu2—O10	89.73 (8)	C3—C8—H8C	109.00
Cu1—O1—C1	120.95 (19)	H8A—C8—H8B	109.00
Cu2—O2—C1	122.11 (18)	H8A—C8—H8C	109.00
Cu1—O3—C9	123.09 (19)	H8B—C8—H8C	109.00
Cu2—O4—C9	120.24 (16)	C11—C12—H12	119.00
Cu1—O5—C17	123.77 (19)	C13—C12—H12	119.00
Cu2—O6—C18	127.16 (19)	C12—C13—H13	120.00
Cu1—O7—C19	121.75 (15)	C14—C13—H13	120.00
Cu2—O8—C19	122.5 (2)	C13—C14—H14	121.00
Cu1—O9—C27	122.8 (2)	C15—C14—H14	121.00
Cu2—O10—C27	121.48 (19)	C10—C15—H15	119.00
C17—O5—H5A	109 (3)	C14—C15—H15	119.00
Cu1—O5—H5A	125 (3)	C11—C16—H16A	110.00
Cu2—O6—H6A	115 (3)	C11—C16—H16B	110.00
C18—O6—H6A	113 (3)	C11—C16—H16C	109.00
O1—C1—O2	124.7 (3)	H16A—C16—H16B	109.00
O1—C1—C2	118.9 (3)	H16A—C16—H16C	109.00
O2—C1—C2	116.4 (3)	H16B—C16—H16C	109.00
C1—C2—C7	117.2 (3)	O5—C17—H17A	110.00
C3—C2—C7	119.9 (3)	O5—C17—H17B	109.00
C1—C2—C3	122.9 (3)	O5—C17—H17C	109.00
C4—C3—C8	119.4 (3)	H17A—C17—H17B	109.00
C2—C3—C4	117.1 (3)	H17A—C17—H17C	110.00
C2—C3—C8	123.4 (3)	H17B—C17—H17C	109.00
C3—C4—C5	121.7 (3)	O6—C18—H18A	109.00
C4—C5—C6	121.1 (4)	O6—C18—H18B	109.00
C5—C6—C7	119.4 (4)	O6—C18—H18C	109.00
C2—C7—C6	120.7 (3)	H18A—C18—H18B	109.00
O3—C9—O4	125.0 (2)	H18A—C18—H18C	109.00
O3—C9—C10	118.0 (3)	H18B—C18—H18C	109.00
O4—C9—C10	116.9 (2)	C21—C22—H22	118.00
C9—C10—C11	122.2 (2)	C23—C22—H22	118.00
C11—C10—C15	120.3 (3)	C22—C23—H23	120.00
C9—C10—C15	117.5 (3)	C24—C23—H23	120.00
C10—C11—C12	116.7 (3)	C23—C24—H24	121.00
C10—C11—C16	123.1 (3)	C25—C24—H24	121.00
C12—C11—C16	120.1 (3)	C20—C25—H25	119.00
C11—C12—C13	122.3 (4)	C24—C25—H25	119.00
C12—C13—C14	120.3 (3)	C21—C26—H26A	109.00
C13—C14—C15	118.8 (3)	C21—C26—H26B	109.00
C10—C15—C14	121.5 (3)	C21—C26—H26C	109.00
O7—C19—C20	119.7 (2)	H26A—C26—H26B	109.00
O8—C19—C20	116.2 (3)	H26A—C26—H26C	109.00
O7—C19—O8	124.1 (3)	H26B—C26—H26C	109.00
C19—C20—C21	123.8 (3)	C29—C30—H30	119.00
C19—C20—C25	116.6 (2)	C31—C30—H30	119.00
C21—C20—C25	119.6 (3)	C30—C31—H31	120.00
C20—C21—C22	116.9 (3)	C32—C31—H31	120.00
C22—C21—C26	120.0 (3)	C31—C32—H32	120.00
C20—C21—C26	123.0 (3)	C33—C32—H32	120.00
C21—C22—C23	123.3 (3)	C28—C33—H33	120.00
C22—C23—C24	119.7 (4)	C32—C33—H33	120.00
C23—C24—C25	118.5 (4)	C29—C34—H34A	109.00
C20—C25—C24	122.1 (3)	C29—C34—H34B	109.00
O10—C27—C28	118.9 (3)	C29—C34—H34C	109.00
O9—C27—O10	124.5 (3)	H34A—C34—H34B	109.00
O9—C27—C28	116.7 (3)	H34A—C34—H34C	109.00
C27—C28—C33	117.2 (3)	H34B—C34—H34C	109.00
			
O3—Cu1—O1—C1	−68.3 (2)	C7—C2—C3—C4	0.4 (4)
O5—Cu1—O1—C1	−164.0 (2)	C7—C2—C3—C8	176.4 (3)
O7—Cu1—O1—C1	98.4 (2)	C1—C2—C7—C6	−178.9 (3)
O1—Cu1—O3—C9	98.7 (2)	C3—C2—C7—C6	1.5 (4)
O5—Cu1—O3—C9	−167.4 (2)	C2—C3—C4—C5	−1.6 (5)
O9—Cu1—O3—C9	−71.0 (2)	C8—C3—C4—C5	−177.7 (4)
O1—Cu1—O5—C17	57.9 (3)	C3—C4—C5—C6	0.8 (6)
O3—Cu1—O5—C17	−31.9 (3)	C4—C5—C6—C7	1.1 (6)
O7—Cu1—O5—C17	146.0 (3)	C5—C6—C7—C2	−2.3 (5)
O9—Cu1—O5—C17	−124.0 (3)	O3—C9—C10—C11	−39.6 (4)
O1—Cu1—O7—C19	−69.7 (2)	O3—C9—C10—C15	139.8 (3)
O5—Cu1—O7—C19	−163.4 (2)	O4—C9—C10—C11	143.2 (3)
O9—Cu1—O7—C19	100.5 (2)	O4—C9—C10—C15	−37.4 (4)
O3—Cu1—O9—C27	95.7 (2)	C9—C10—C11—C12	178.1 (3)
O5—Cu1—O9—C27	−168.4 (2)	C9—C10—C11—C16	−5.0 (4)
O7—Cu1—O9—C27	−70.7 (2)	C15—C10—C11—C12	−1.3 (4)
O4—Cu2—O2—C1	104.1 (2)	C15—C10—C11—C16	175.6 (3)
O6—Cu2—O2—C1	−164.4 (2)	C9—C10—C15—C14	−176.3 (3)
O8—Cu2—O2—C1	−66.7 (2)	C11—C10—C15—C14	3.1 (5)
O2—Cu2—O4—C9	−66.4 (2)	C10—C11—C12—C13	−1.6 (5)
O6—Cu2—O4—C9	−162.3 (2)	C16—C11—C12—C13	−178.6 (3)
O10—Cu2—O4—C9	100.6 (2)	C11—C12—C13—C14	2.7 (5)
O2—Cu2—O6—C18	150.2 (3)	C12—C13—C14—C15	−0.8 (5)
O4—Cu2—O6—C18	−121.4 (3)	C13—C14—C15—C10	−2.0 (5)
O8—Cu2—O6—C18	60.8 (3)	O7—C19—C20—C21	−35.8 (5)
O10—Cu2—O6—C18	−30.0 (3)	O7—C19—C20—C25	143.3 (3)
O2—Cu2—O8—C19	96.3 (2)	O8—C19—C20—C21	146.1 (3)
O6—Cu2—O8—C19	−167.91 (19)	O8—C19—C20—C25	−34.8 (4)
O10—Cu2—O8—C19	−70.8 (2)	C19—C20—C21—C22	179.2 (3)
O4—Cu2—O10—C27	−70.4 (2)	C19—C20—C21—C26	−2.8 (5)
O6—Cu2—O10—C27	−162.2 (2)	C25—C20—C21—C22	0.2 (5)
O8—Cu2—O10—C27	100.0 (2)	C25—C20—C21—C26	178.2 (3)
Cu1—O1—C1—O2	−5.1 (4)	C19—C20—C25—C24	−178.1 (3)
Cu1—O1—C1—C2	175.2 (2)	C21—C20—C25—C24	1.0 (5)
Cu2—O2—C1—O1	−13.4 (4)	C20—C21—C22—C23	−1.1 (6)
Cu2—O2—C1—C2	166.3 (2)	C26—C21—C22—C23	−179.1 (4)
Cu1—O3—C9—O4	−4.6 (4)	C21—C22—C23—C24	0.8 (6)
Cu1—O3—C9—C10	178.5 (2)	C22—C23—C24—C25	0.5 (6)
Cu2—O4—C9—O3	−12.2 (4)	C23—C24—C25—C20	−1.3 (6)
Cu2—O4—C9—C10	164.7 (2)	O9—C27—C28—C29	152.6 (3)
Cu1—O7—C19—O8	−11.6 (4)	O9—C27—C28—C33	−26.3 (4)
Cu1—O7—C19—C20	170.5 (2)	O10—C27—C28—C29	−27.7 (5)
Cu2—O8—C19—O7	−5.2 (4)	O10—C27—C28—C33	153.3 (3)
Cu2—O8—C19—C20	172.75 (19)	C27—C28—C29—C30	−177.4 (3)
Cu1—O9—C27—O10	−5.8 (4)	C27—C28—C29—C34	0.3 (6)
Cu1—O9—C27—C28	173.90 (19)	C33—C28—C29—C30	1.5 (5)
Cu2—O10—C27—O9	−10.5 (4)	C33—C28—C29—C34	179.2 (4)
Cu2—O10—C27—C28	169.8 (2)	C27—C28—C33—C32	178.6 (4)
O1—C1—C2—C3	−34.3 (4)	C29—C28—C33—C32	−0.4 (6)
O1—C1—C2—C7	146.2 (3)	C28—C29—C30—C31	−1.2 (6)
O2—C1—C2—C3	146.0 (3)	C34—C29—C30—C31	−179.1 (4)
O2—C1—C2—C7	−33.6 (4)	C29—C30—C31—C32	−0.1 (7)
C1—C2—C3—C4	−179.2 (3)	C30—C31—C32—C33	1.3 (7)
C1—C2—C3—C8	−3.2 (4)	C31—C32—C33—C28	−1.0 (7)

Symmetry codes: (i) −*x*+1, −*y*, −*z*; (ii) −*x*+1, −*y*, −*z*+1; (iii) −*x*+1, −*y*+1, −*z*+1; (iv) *x*−1, *y*, *z*; (v) *x*+1, *y*, *z*; (vi) −*x*+1, −*y*−1, −*z*; (vii) *x*, *y*−1, *z*; (viii) *x*, *y*+1, *z*.

### Hydrogen-bond geometry (Å, °)

**Table:**

Cg1 and Cg2 are the centroids of C2–C7 and C28–C33 rings, respectively.

**Table:**

*D*—H···*A*	*D*—H	H···*A*	*D*···*A*	*D*—H···*A*
O5—H5A···O7^i^	0.70 (3)	2.11 (3)	2.793 (3)	167 (4)
O6—H6A···O2^ii^	0.72 (3)	2.10 (3)	2.812 (2)	174 (4)
C18—H18A···Cg1^ii^	0.96	2.98	3.749 (4)	137.00
C23—H23···Cg1^vi^	0.93	2.87	3.735 (4)	154.00
C13—H13···Cg2^iii^	0.93	2.97	3.877 (4)	165.00

Symmetry codes: (i) −*x*+1, −*y*, −*z*; (ii) −*x*+1, −*y*, −*z*+1; (vi) −*x*+1, −*y*−1, −*z*; (iii) −*x*+1, −*y*+1, −*z*+1.
